# Effect of Cortical Bone Thickness on Detection of Intraosseous Lesions by Ultrasonography

**DOI:** 10.1155/2015/797593

**Published:** 2015-08-23

**Authors:** Sadaf Adibi, Alireza Shakibafard, Zohreh Karimi Sarvestani, Najmeh Saadat, Leila Khojastepour

**Affiliations:** ^1^Dentomaxillofacial Department, Faculty of Dentistry, Shiraz University of Medical Sciences, Shiraz, Iran; ^2^TABA Radiology Center, Shiraz, Iran; ^3^Faculty of Dentistry, Shiraz University of Medical Sciences, Shiraz, Iran

## Abstract

*Background*. Usefulness of ultrasound (US) in detection of intrabony lesions has been showed. A cortical bone perforation or a very thin and intact cortical bone is prerequisite for this purpose.* Objective*. The current in vitro study was aimed at measuring the cut-off thickness of the overlying cortical bone which allows ultrasonic assessment of bony defects.* Materials and Methods*. 20 bovine scapula blocks were obtained. Samples were numbered from 1 to 20. In each sample, 5 artificial lesions were made. The lesions were made in order to increase the overlying bone thickness, from 0.1 mm in the first sample to 2 mm in the last one (with 0.1 mm interval). After that, the samples underwent ultrasound examinations by two practicing radiologists.* Results*. All five lesions in samples numbered 1 to 11 were detected as hypoechoic area. Cortical bone thickness more than 1.1 mm resulted in a failure in the detection of central lesions.* Conclusion*. We can conclude that neither bony perforation nor very thin cortical bones are needed to consider US to be an effective imaging technique in the evaluation of bony lesion.

## 1. Introduction

Since the first set of data of diagnostic ultrasound (US) was reported in dentistry by Baum et al. [1] in 1963, many new different ultrasound applications have been conducted. Detecting carious lesions, dental fractures or cracks, soft tissue lesions, maxillofacial fractures, periodontal bony defects, measurement of muscle and gingival thickness, diagnosis of temporomandibular disorders, implant dentistry, and dental scanning by ultrasound (US) have been a primary subject for recent studies [2–11]. Although ultrasonography is primarily used for soft tissue imaging, many studies have shown the utility of this technique in the detection of intrabony lesions [12], monitoring the healing of periapical lesions [6], and the differential diagnosis of periapical lesions [13]. US and Doppler imaging have also provided specific information regarding the size and the nature of intrabony lesions, without any radiation risk [6]. Furthermore, these reproducible, convenient, and straightforward techniques allow for the evaluation of the presence, nature, and velocity of blood flow in the examined tissue. Also, the formation of new vessels in bone during the initial healing period can be revealed by this technique [14]. As bone surface reflects ultrasound completely, structures in and beyond intact bone are not normally detectable by ultrasonography [15]. Tikku et al. [6] in agreement with Gundappa et al. [15] and Rajendran and Sundaresan [16] reported that the prerequisite for detecting and evaluating the central lesions of the jaw is a breach or perforation in the buccal bone plate. However, according to Raghav et al. [17] and Aggarwal and Singla [18], where the overlying bone has become thinned, ultrasonography imaging can still be performed through such bone windows. Therefore, there is controversy over the need for an element for the evaluation of the intrabony lesions. In the current academic literature, there is no reported study on the thickness of the cortical bone which masks the underlying lesion. Accordingly, this study was designed to determine the thickness of cortical bone which reflects the ultrasound waves completely and results in a failure in the detection of bony lesions.

## 2. Materials and Methods

**Figure 1:**
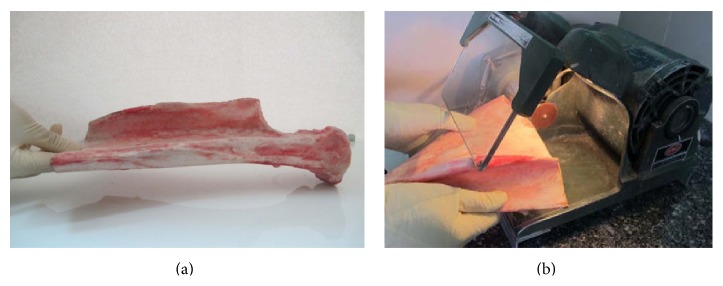
Bovine scapula. (a) Ventral surface. (b) The flat part is separated by dental disc and cutting Lathe motor machine.

20 blocks were harvested from flat part of the body of the bovine scapula (Figure 1(a)) obtained from the local slaughter of twelve healthy one-year-old bulls. The soft tissue was removed from the scapula prior to cutting. The samples were preserved in formaldehyde.

**Figure 2:**
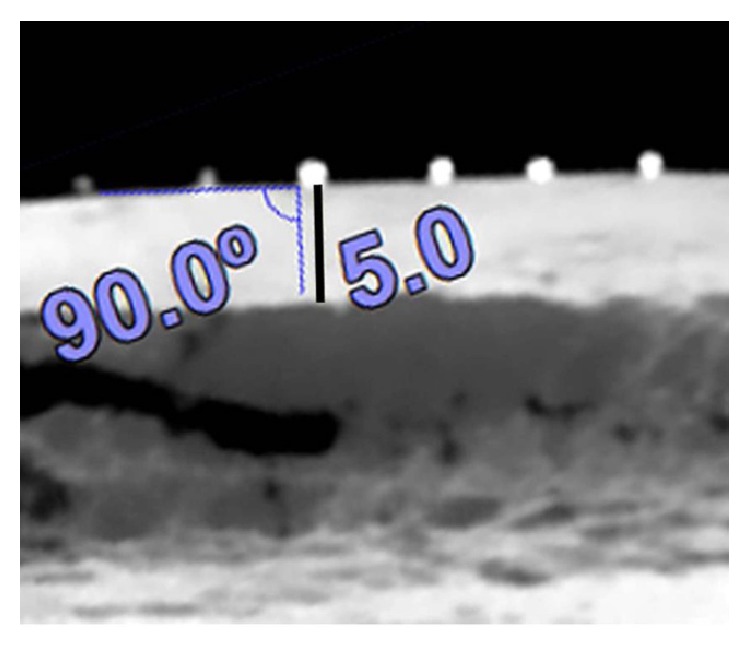
At right angle to the bone surface (90°) the thickness of cortical point beneath the gutta percha is 5 mm.

**Figure 3:**
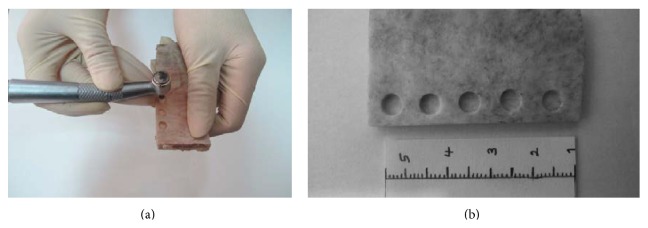
(a) The holes are made by wheel bur and high speed hand piece. (b) All the lesions are 5 mm in diameter.

**Figure 4:**
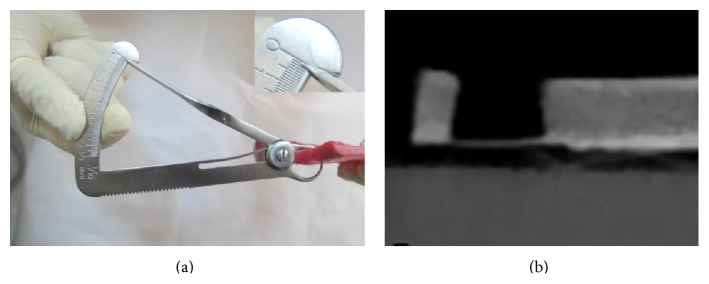
(a) The remaining cortical bone was measured by dental caliper (here in sample number 1 the thickness is 0.1 mm). (b) In cross-sectional view of CBCT image, no perforation is found.

**Figure 5:**
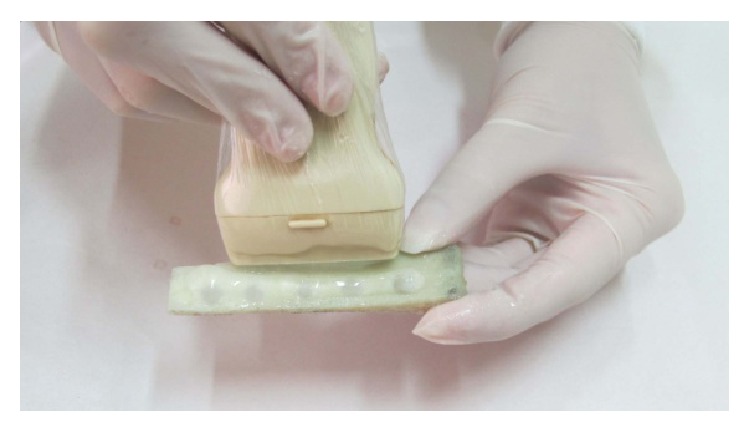
Ultrasonic gel was applied on the intact surface on the linear ultrasonic probe that was placed on the block.

The blocks were cut using a high speed dental cutting Lathe motor machine (Figure 1(b)). The surfaces of the samples were marked by 0.5-millimeter pieces of gutta percha (size 40), with 5-millimeter interspace. The blocks got fixed by wax on the chin rest of the CBCT machine and images were taken (by Newtom VGI, QR srl, Italy). In the cross-sectional view, the cortical bone thickness was measured beneath each marked point for determining the points with more than 2-millimeter overlay (Figure 2). The bone samples were numbered from 1 to 20. In each bone sample, five artificial lesions were made using a high speed NSK hand piece and 818-diamond wheel bur (Figure 3(a)). Each lesion had a diameter of 5 millimeters and was located beneath the marked points (Figure 3(b)). Considering 0.1 mm intervals, the overlying cortical bone thickness was increased from 0.1 mm in the first sample (number 1) to 2 mm in the last one (number 20). A Dental Crown Gauge Caliper, with an accuracy of 0.1 millimeter, was used for the measurement of the remaining cortical bone (Figure 4(a)). CBCT images from all samples were obtained to ensure that there are no perforations (Figure 4(b)). Next, the bony blocks underwent ultrasound evaluation by two experienced radiologists. The images were analyzed, at the same time that the examination was done, by two observers so a mutual agreement about the presence of the lesion could be made. Medison US equipment (V20 Prestige, Seoul, Korea) was used for this study. The images were obtained at 6–12 MHz by a 40-millimeter, linear probe. The probe was first covered with disposable latex and then covered with a layer of ultrasound gel; also the surfaces of the samples were covered with ultrasonic gel. The probe was positioned on the intact surface of each block in order to find the lesions (Figure 5). The positions of the probe and ultrasonic gain and mode (per, gen, and res) were changed several times in order to obtain high quality scans. At each step of the protocol, the investigators were blind to the overlying bone thickness and the similarity of the lesions in each block.

## 3. Results

**Figure 6:**
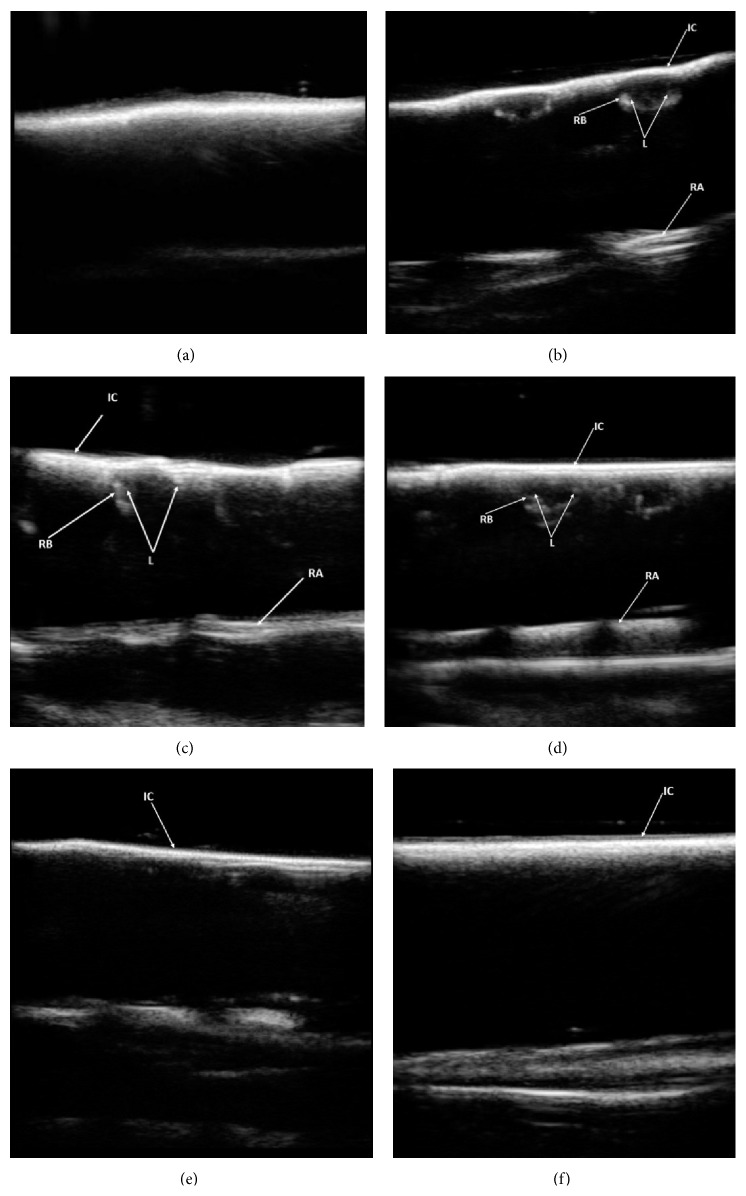
(a) Ultrasonogram of intact bone without any lesion. Ultrasonograms of sample: (b) number 1, (c) number 2, (d) number 11, (e) number 12, and (f) number 20. IC (intact cortical bone), RB (remaining bone between cavities), RA (reverberation artifact), and L (lesion).

55 out of 100 intrabony simulated lesions were detected by ultrasonographic examinations. All the artificial lesions in blocks 1 to 11 were detected (Figure 6). They appeared as poorly defined hypoechoic areas beneath hyperechoic overlying cortices, compared to the adjacent normal bone. None of the lesions were detected in blocks 12 to 20 (Table 1).

## 4. Discussion

The use of ultrasound in inflammatory soft tissue conditions and superficial tissue disorders of the head and neck region has been long established [19]. In spite of all limitations, monitoring for bone healing and the detection of intrabony lesions by US have shown promising results [1]. This study has assessed the maximum thickness of the cortical bone which interferes with the ultrasonic evaluation of intrabony lesions. In order to decrease the rate of accidental finding of the lesions, five cavities were made in each bony block. Our results are in agreement with the findings of Dib et al. [20], Gundappa et al. [15], Raghav et al. [17], Prince et al. [21], and Maity et al. [22] and in contrast with those of Aggarwal et al. [18, 23], Tikku et al. [6], Cotti et al. [13], Rajendran and Sundaresan [16], and Goel et al. [24], who have reported that ultrasound cannot penetrate and diagnose the presence of an intrabony lesion unless there is a breach on the overlying bone plate. Based on this study, the cut-off point of cortical bone thickness that masks the intrabony lesions was measured at 1.1 mm. Currently, in academic literature, there is no reported study on the thickness of cortical bone which conceals the intrabony lesions.

When ultrasound bounces between two interfaces with high acoustic impedance, it will move forward and backward between these interfaces. These waves will be shown as parallel lines which are called reverberation artifact. Furthermore, objects beyond these interfaces usually cannot be imaged through US [25]. In our samples, the cortical bone was intact, so this artifact could be observed in all blocks as hyperechoic interrupted lines (Figures 6(b), 6(c), and 6(d)). However, in those sites, where the cortex had been thinned, hypoechoic shadows of the lesions could be seen and the hyperechoic areas amongst them relate to the bony septa between the cavities. As accessibility to flat bony superficies in human skeleton is not possible, bovine scapula was used for the preparation of the samples.

In 2008 Wijnhoud et al. [26] studied the relationship between skull thickness and the radiodensity of the temporal bone and window failure. The temporal bone window is the thinnest area of the lateral skull which allows for US beam to transmit. Through this window, transcranial Doppler ultrasonography can be done to evaluate the intracranial blood flow. In some patients, no adequate Doppler signal can be found because of an inadequate acoustic temporal window or overall window failure. In this study, they concluded that skull thickness is more closely related to window failure compared to the radiodensity of the temporal bone. Considering this conclusion, it seems that different bone densities do not alter the result of our study. However to remove the probable effect of bone density on accuracy of ultrasonographic examination, five lesions with similar diameter and similar thickness of the remaining cortical bone were prepared in each block. If lesions were made in different samples, it would not be clear that the cause of failure in detection is differed bone density or thickness.

According to Alves et al. [27] the bovine bone has a higher density and a higher acoustic impedance compared to human samples. Therefore, ultrasound waves are reflected more when interacted with the bovine bone. So, it is expected that, in human samples, a thicker cortical bone (more than 1.1 mm) reflects the ultrasound waves completely and therefore camouflages the underlying intrabony lesions.

The ages of the bulls were not considered in the previous studies done on the characteristics of ultrasound waves in the bovine bone [28–30]. Even so, to omit the probable effect of bone density on ultrasound detection accuracy, we used one-year-old bulls.

Federspil et al. [19], in 2010, measured the temporal bone thickness by ultrasonography. They used formaldehyde-preserved human cadaver temporoparietal bones and the ultrasonic probe was placed directly on the bone surface with no soft tissue or soft tissue substitute being used. Similarly, in the present study, the soft tissues were removed in order to measure the bone thickness by a dental caliper.

Since digital or computer controlled items of equipment were not available to make the cavities, 5 mm wheel bur size was used to keep the uniformity of diameter and shape in all the lesions. In the ultrasonograms of intraosseous lesions, an acoustic shadow is casted over the lateral walls of the lesions, making it difficult to locate the margins precisely [15]. So if the lesions in this study had a larger diameter, they might be detected even beyond the remaining thicker cortical plate (more than 1.1 mm).

All in all, further research is recommended to investigate the effect of lesion size on the capability of US to detect intrabony lesions. Due to the limitations of the current study, it is highly suggested that future studies use different human samples and ultrasonographic machines.

Since even a 5 mm bony lesion is detected under a relatively thick cortical bone, it can be concluded that there is no necessity for very thin or perforated cortical bone to detect intraosseous lesions including cysts and tumors, by ultrasound. So we suggest the use of US as a potential diagnostic tool for the assessment of central lesions even when there is no perforated cortex.

## Figures and Tables

**Table 1:** Number of detected cavities in different bone thicknesses.

Bone thickness mm	Lesions	
	Simulated *n*	Detected *n* (%)
0.1	5	5 (100%)
0.2	5	5 (100%)
0.3	5	5 (100%)
0.4	5	5 (100%)
0.5	5	5 (100%)
0.6	5	5 (100%)
0.7	5	5 (100%)
0.8	5	5 (100%)
0.9	5	5 (100%)
1	5	5 (100%)
1.1	5	5 (100%)
1.2	5	0 (0%)
1.3	5	0 (0%)
1.4	5	0 (0%)
1.5	5	0 (0%)
1.6	5	0 (0%)
1.7	5	0 (0%)
1.8	5	0 (0%)
1.9	5	0 (0%)
2	5	0 (0%)
